# TeraVR empowers precise reconstruction of complete 3-D neuronal morphology in the whole brain

**DOI:** 10.1038/s41467-019-11443-y

**Published:** 2019-08-02

**Authors:** Yimin Wang, Qi Li, Lijuan Liu, Zhi Zhou, Zongcai Ruan, Lingsheng Kong, Yaoyao Li, Yun Wang, Ning Zhong, Renjie Chai, Xiangfeng Luo, Yike Guo, Michael Hawrylycz, Qingming Luo, Zhongze Gu, Wei Xie, Hongkui Zeng, Hanchuan Peng

**Affiliations:** 1 Southeast University – Allen Institute Joint Center, Institute for Brain and Intelligence, Southeast University, Nanjing, 210096 China; 2 School of Computer Engineering and Science, Shanghai University, Shanghai, 200444 China; 3 Shanghai Institute for Advanced Communication and Data Science, Shanghai University, Shanghai, 200444 China; 4 Allen Institute for Brain Science, Seattle, 98109 USA; 5 School of Optometry and Ophthalmology, Wenzhou Medical University, Wenzhou, 325027 China; 6 Beijing University of Technology, 100124, Beijing, China; 7 Department of Life Science and Informatics, Maebashi Institute of Technology, Maebashi, 371-0816 Japan; 8 Institute of Life Sciences, Southeast University, Nanjing, 210096 China; 9 Key Laboratory for Developmental Genes and Human Disease, Ministry of Education, Institute of Life Sciences, Jiangsu Province High-Tech Key Laboratory for Bio-Medical Research, Southeast University, Nanjing, 210096 China; 10 Co-Innovation Center of Neuroregeneration, Nantong University, Nantong, 226019 China; 11 Data Science Institute, Imperial College London, London, SW7 2AZ UK; 12 Britton Chance Center for Biomedical Photonics, Wuhan National Laboratory for Optoelectronics, Huazhong University of Science and Technology, Wuhan, 430074 China; 13 School of Biological Science and Medical Engineering, Southeast University, Nanjing, 210096 China

**Keywords:** Neuroscience, Computational biology and bioinformatics

## Abstract

Neuron morphology is recognized as a key determinant of cell type, yet the quantitative profiling of a mammalian neuron’s complete three-dimensional (3-D) morphology remains arduous when the neuron has complex arborization and long projection. Whole-brain reconstruction of neuron morphology is even more challenging as it involves processing tens of teravoxels of imaging data. Validating such reconstructions is extremely laborious. We develop TeraVR, an open-source virtual reality annotation system, to address these challenges. TeraVR integrates immersive and collaborative 3-D visualization, interaction, and hierarchical streaming of teravoxel-scale images. Using TeraVR, we have produced precise 3-D full morphology of long-projecting neurons in whole mouse brains and developed a collaborative workflow for highly accurate neuronal reconstruction.

## Abstract (web summary)

Reconstructing the full shape of neurons is a major informatics challenge as it requires handling huge whole-brain imaging datasets. Here the authors present an open-source virtual reality annotation system for precise and efficient data production of neuronal shapes reconstructed from whole brains.

## Introduction

Major international initiatives are underway to profile and characterize cell types of the mammalian brain^1,2^. As a key recognized attribute of cell type since Ramon y Cajal, high-fidelity reconstruction of neuron morphology is gaining increased attention^3–5^. The basic building blocks of the brain, neurons and glial cells, are often noted for their remarkable three-dimensional (3-D) shapes that distinguish one cell type from another. While such shapes are critical to understanding cell type, function, connectivity, and development^6^, it is challenging to profile these shapes precisely. Sparse labeling and high-resolution micro-imaging of a brain cell help visualize the appearance of the cell, yet it remains a major bottleneck how to convert such imaging data into a digital description of morphology, including the 3-D spatial locations of a cell’s parts and their topological connections. This conversion process is often called neuron tracing or neuron reconstruction and it has become an essential and active area of neuroinformatics.

Two complementary reconstruction workflows exist: one for electron microscopy (EM) images and the other for light microscopy (LM) data^7–9^. EM offers nanometer resolution and thus provides a way to reconstruct the entire surface of the shape, but it is often constrained to relatively small brain regions. When whole-brain scale is the focus and complete neuron morphology is desired, LM is a more suitable imaging modality where data are typically acquired at sub-micrometer resolution. LM reconstruction makes it possible to trace both long projections and the terminal arborization of a brain cell. Recent extension of this approach based on expansion microscopy can help visualize neurons at nanometer resolution using LM approaches^10^.

It is widely recognized that manual and semi-automatic neuron-tracing methods are crucially required to produce full reconstructions, which can also serve as gold-standard datasets to develop fully automatic neuron-tracing methods^9,11–14^. Without loss of generality below, we define any neuron-tracing method that has a non-negligible human labor component as manual reconstruction, which clearly also includes many semi-automatic methods. This paper discusses a technology that makes such LM-oriented manual reconstruction more efficient and reliable than existing approaches. This work was motivated by four difficulties detailed below: (1) observability, (2) big data handling, (3) interaction, and (4) validation.

First, a neuron can have a very complex 3-D shape that may contain hundreds or even thousands of fiber branches, especially in dense arbors. Such a high degree of mutual occlusion makes it hard to see how neurite fibers wire together. The observability is further compromised by the uneven or weak axon labeling, relatively poor Z-resolution from imaging, and so on. Often, neither the prevailing 2-D cross-sectional view (such as those widely used in EM-oriented and many LM software packages) nor the typical 3-D intensity projection methods^15^ are sufficient to unambiguously delineate these complex wiring patterns, let alone reconstruct them.

Second, reconstructing the full morphology of a mammalian neuron relies on effectively managing and streaming huge whole-brain imaging datasets. The volume of a typical mouse brain is about 500 mm^3^, it is not uncommon that a neuron may have over 100-ml-long neurite fiber^5^. When an entire mouse brain is imaged at sub-micrometer resolution in 3-D, the volume of the acquired brain images often contains 20 to 30 or more teravoxels. Only a small number of existing software packages are able to open and analyze such big datasets^16,17^. How to streamline the unambiguous 3-D visualization and analysis of such huge datasets presents a major informatics challenge.

Third, manual reconstruction of neurons is often laborious and unintuitive using two-dimensional (2-D) tools to interact with 3-D images and the 3-D geometrical representations reconstructed from such images. Reconstructing geometrical objects from 3-D volumetric images requires overlaying these objects onto the imaging data in 3-D space and manipulating them in situ. Since most current computer displays (e.g., computer screens) and data interaction tools (e.g., computer mouse) are still restricted to 2-D, it is usually hard to observe and manipulate higher dimensional data via a lower dimensional interface. It is also desirable to interact with the data directly using a smooth workflow. Applications such as Virtual Finger^18^ represent progress toward this goal, but improvement is still necessary for complex and large neurons and also for display and interaction hardware.

Finally, it is often necessary but very expensive to involve multiple annotators to produce gold-standard reconstructions. Manual work is time-consuming and tedious, thus in practice most existing studies can afford only one annotator per neuron. To resolve any ambiguity of reconstructions, it is desired to have a way to allow multiple annotators to visualize the same neuron and its underlying imaging data at the same time, and collaborate on the work. This approach requires collaborative and immersive annotation of multi-dimensional imaging data at the whole-brain scale.

Here we consider using virtual reality (VR) techniques. While there have been commercial VR systems (e.g., arivisVR) and research software ^19,20^, it is not straightforward to use any of these existing work to tackle the above challenges. We introduce the TeraVR system addressing the above requirements. We demonstrate the applicability of TeraVR to challenging cases of whole mouse brain neuron reconstruction, achieving previously unattainable accuracy and efficiency.

## Results

### TeraVR platform

**Fig. 1:**
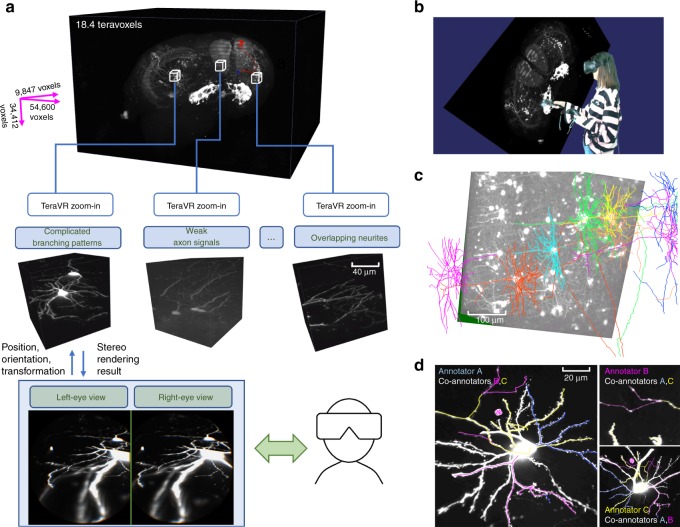
The overall scheme of TeraVR. **a** TeraVR is applicable to very challenging visualization and reconstruction scenarios such as complicated branching, weak signals, and overlapping neurites. With TeraVR, a user is able to combine stereoviews to observe the complex 3-D neurite patterns easily and perform the reconstruction effectively. Combining such visualization and data-exploration functions with terabyte-scale imaging data (e.g., whole-brain scale) management and streaming capability enables reconstruction of complex neuronal morphology at an optimized accuracy and efficiency. **b** A mixed reality visualization that demonstrates the use of TeraVR. Immersed in a virtual environment, the user manipulates the imaging data with TeraVR in a way similar to manipulating a physical object. **c** Multiple densely packed neurons (one in a different color) from an image with high, noisy background intensity level were reconstructed using TeraVR. **d** Real-time collaboration is demonstrated by showing views from all participating annotators. Each annotator logs onto the cloud and adopts a unique color for both annotation and an avatar representing the user’s real-time location. The left subpanel shows the view for annotator A (blue), in which two avatars of co-annotator B (purple) and C (yellow) are seen. Annotation results are instantly shared among them. The upper right subpanel: annotator B examined a partially traced segment by co-annotator C, only to identify more branches after turning up the contrast and having a close-up view of the segment (without affecting the views of other annotators); bottom right subpanel: the view of annotator C

We developed TeraVR (Fig. 1, Supplementary Note 1, and Supplementary Movies 1–11), an open-source VR software package for the visualization and annotation of teravoxel-scale whole-brain imaging data (Fig. 1a). The software uses the TeraFly^16^ module of Vaa3D (http://vaa3d.org) to manage data input–output (I/O), thus TeraVR can streamline the data I/O and other real-time user interaction with teravoxel-scale image volumes, for example, an 18.4-teravoxel brain image in Fig. 1a. As described below, TeraVR is much more than a simple extension of TeraFly. Indeed, it has a number of unique features designed for reconstruction of neuron morphology in whole-brain images, at different levels of details and at different local regions of interest (ROI).

To use TeraVR, a user wears a VR headset (bottom right of Fig. 1a) and works within a virtual space defined for the brain image along with the neuron reconstruction and other location references on the image. TeraVR generates synchronized real-time rendering streams for both left and right eyes (bottom left of Fig. 1a), which simulate how a person perceives real-world objects and thus forms stereo vision. In this way, TeraVR facilitates efficient immersive observation and annotation (Fig. 1b and Supplementary Movie 12) of very large-scale multi-dimensional imaging data, which can have multiple channels or from different imaging modalities (Supplementary Fig. 1). With the accurate pinpointing capability in TeraVR (Supplementary Fig. 2), in real time a user can precisely and efficiently load the data of a desired high-resolution ROI to see detailed 3-D morphological structures (Supplementary Fig. 2c). For a neuroanatomist, the immersive 3-D environment of TeraVR enables a user to observe and infer the complex 3-D trajectories of neurites much more easily. The data handling of TeraVR has been engineered to be scalable so that the large amount of volumetric data is no longer a barrier. In addition, a comprehensive set of assisting functions such as convenient contrast and display modes adjusting, whole-brain-wide orientation and navigation, adding/editing/removing of 3-D geometrical objects, automatic tract-signal alignment, and so on, have all been made available in TeraVR in an ergonomic way. Many of the typical usages of TeraVR can be found in the Supplementary Movies 1–12.

The whole-brain imaging data typically contains complicated branching patterns, weak and discontinuous axon signals, overlapping neurites, and so on (middle of Fig. 1a). A user employs TeraVR to gain unambiguous understanding on a considerable number of such challenging regions that are otherwise very hard, if not impossible, to distinguish confidently using any existing non-immersive visualization tools. TeraVR provides comprehensive tools for neuron reconstruction. In addition to single neurons, TeraVR was also used to reconstruct multiple densely packed neurons in very noisy images (Fig. 1c). TeraVR also allows multiple annotators working on the same dataset collaboratively using a cloud-based data server (Fig. 1d), in a way similar to Google Docs, to combine multiple users’ input together efficiently.

### Efficient tracing using TeraVR

**Fig. 2:**
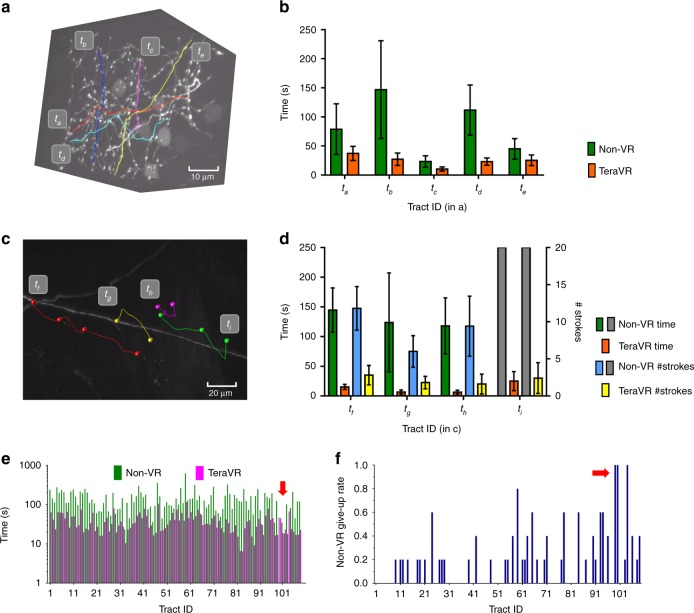
Efficiency of TeraVR. **a** A complex three-dimensional (3-D) image volume with a number of intermingled, broken, strongly punctuated axon tracts *t*_a_ ~ *t*_e_. **b** Time spent to generate the five tracts in **a**, each of which was produced by five independent annotators; the “non-virtual reality (VR)” results showed were obtained using TeraFly (same below in this figure); error bar: SD. **c** A 3-D image volume with weak signal and strong noise, and the respective TeraVR reconstructions of barely visible neurite tracts *t*_f_ ~ *t*_i_. **d** Time and the number of operations needed to produce the tracts in **c**. Gray bar: unavailable results (time/number of strokes) for non-VR approach. **e** Average time of 5 annotators to generate 109 tracts, which were hard to reconstruct. For non-VR, the average was calculated among the sub-group of annotators who succeeded in reconstructing the tract. **f** The give-up rate of non-VR for each tract in **e**; an annotator was allowed to give up the attempt after trying 300 s; the give-up rate for each tract was defined as (#failed attempts)/(#all attempts). Arrows in **e**, **f**: the cases where no non-VR attempt was able to produce the respective neurite tracts

We tested TeraVR in challenging situations for conventional non-VR approaches due to densely labeled and weakly imaged neurites. Such non-VR approaches include many visualization and annotation functions already existing in Vaa3D and TeraFly, as well as in other software packages such as ImageJ/Fiji (https://fiji.sc/) and Neurolucida (MBF Bioscience). First, for a strongly punctuated and highly intermingled axon cluster (Fig. 2a), five independent annotators reduced the time in tracing by 50–80% when they used TeraVR compared to TeraFly, the most efficient non-VR approach we found for these testing cases (Fig. 2b). Second, for exceedingly weak neurite signals (Fig. 2c), with TeraVR these annotators could consistently generate a neurite tract (bounded by branching points and/or terminal points) within 50 s, about 10 times faster than the non-VR approach (Fig. 2d). For these weak signals, even when sometimes annotators needed to adjust the contrast in the visualization in both TeraVR and non-VR approaches, it was much easier for the annotators to use TeraVR than the non-VR method to find the right angle of observation and to add annotations on top of the signals. TeraVR reduced 60–80% of labor when measured with alternative metrics such as the number of strokes to complete a neurite tract in d–rawing (Fig. 2d). We also examined the speed of annotation done for the nine tracts in Fig. 2a, c and observed that TeraVR was much faster than the non-VR approaches (Supplementary Fig. 3) in all test cases. Third, for 109 dense or weak tracts, with TeraVR these annotators rarely needed more than 50 s to reconstruct any of such difficult tracts, while the non-VR approach normally needed about 10 times of effort for the same task (Fig. 2e). In 37.6% of tracts in this testing set, at least one annotator was not able to use the non-VR approach to reconstruct (Fig. 2f), while none of these annotators had trouble to accomplish the goal when TeraVR was used. Finally, in the majority of cases we found that five annotators were able to use TeraVR independently to generate mutually much more consistent reconstructions than using the non-VR approaches (Supplementary Fig. 4).

### Brain-wide neuron reconstruction using TeraVR

A neuron may contain thousands or more neurite tracts, each of which is bounded by a pair of critical points, for example, branching points, axonal or dendritic terminals, or the cell body (soma). Neurites are organized into local dendritic arbors, local axonal arbors, long-projecting axon fibers, and distal axonal arbors. While some structures such as the major dendritic branches may be reconstructed using non-VR approaches, many other challenging cases (e.g., Fig. 2) will require the VR module in TeraVR for faithful and efficient reconstruction. Therefore, in TeraVR we designed a smooth switch between the VR mode and the non-VR mode to allow an annotator to choose a suitable mode to observe the imaging data and reconstruct neurites for different areas in a big imaging dataset.

**Fig. 3:**
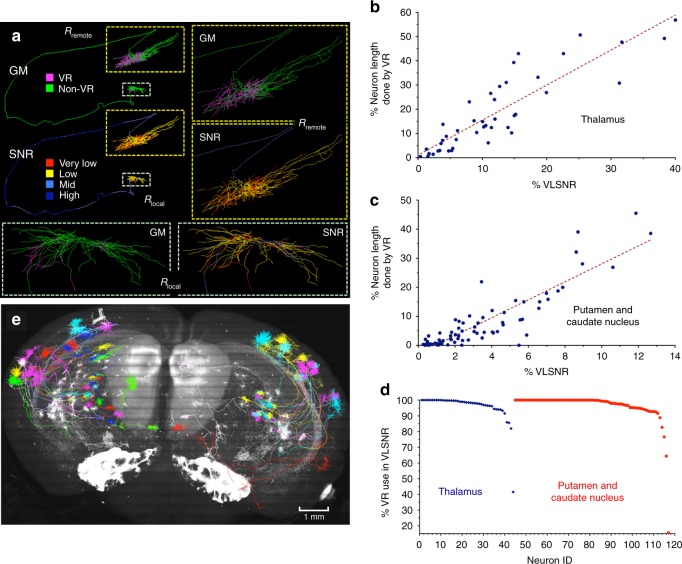
Complete reconstruction of neurons at whole-brain scale using TeraVR. **a** A thalamic cell reconstructed using TeraVR. Upper left: a complete reconstruction of the neuron color-coded using “GM” (Generation Method) and “SNR” (signal-to-noise-ratio) schemes; in “GM,” magenta and green colors stand for neurites reconstructed using virtual reality (VR) and non-VR, respectively; in “SNR,” blue, sky blue, yellow, and red colors indicate neurites with high, mid, low, and very low SNR, respectively; two close-up views of local dendrites and remote axons are also shown in the right and the bottom. **b** For a set of 44 completely reconstructed thalamic neurons (33 from brain no. 17302, 11 from brain no. 17545), the correlation between the portion of a neuron traced using the VR mode of TeraVR and the portion of this neuron that has very low SNR (VLSNR). **c** For a set of 73 completely reconstructed neurons in caudate putamen (58 from brain no. 17302 and 15 from brain no. 17545), the correlation between the portion of a neuron traced using the VR mode of TeraVR and the portion of this neuron that has very low SNR (VLSNR). **d** The use of VR mode in reconstruction of BASNR (below average SNR) regions in each of the 117 neurons. **e** Whole-brain plot of 33 thalamic neurons reconstructed from brain no. 17302; gray: maximal intensity projection of this brain image; color code: each neuron in a randomly assigned color

This technology allowed us to reconstruct complete 3-D morphology of neurons from the whole mouse brain, each of which was repeatedly curated by four to five annotators to ensure accuracy. To better understand the usability of TeraVR, we trained 15 annotators to independently produce complete reconstructions for different types of neurons. During the process, an annotator can flexibly switch between either VR or non-VR mode, depending on the characteristics of the encountered imaging signals. We analyzed under which situations these annotators would switch between VR and non-VR modes to understand the strength of the VR mode (Fig. 3 and Supplementary Fig. 5). VR was used mostly in densely arbored areas such as axonal arbors and sometimes also in local dendrites (Fig. 3a and Supplementary Fig. 5a–c). The areas done by VR often have low or very low signal-to-noise-ratio (SNR) (Fig. 3a, Supplementary Fig. 5, and Methods). For 44 thalamic neurons in two mouse brains, the percentage of very low SNR regions correlated linearly with the VR portion of neurons (Fig. 3b). Linear correlation was also observed in analyzing 73 neurons in caudate putamen in the two brains (Fig. 3c). For all these 117 neurons together, over 90% of VR usage was dedicated to the reconstruction of neurites in the below average SNR regions (Fig. 3d).

### TeraVR helps improve existing neuron reconstructions

**Fig. 4:**
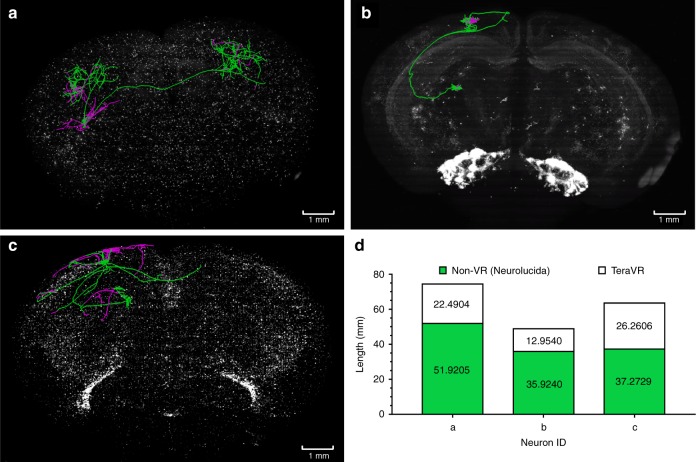
The use of TeraVR in validating, correcting, and extending complex neuron reconstructions produced with Neurolucida. **a**–**c** Three examples of reconstructed neurons overlaid on the whole-mouse brain imaging data, from three different brains (brain no. 236174, 17545, 17300), respectively. Green: initial reconstructions produced using Neurolucida; magenta: recovered missing portion of reconstructions using TeraVR. **d** The length of neuron reconstructions produced for **a**–**c**, respectively

We further investigated whether reconstructions of similar accuracy could have been produced using other commonly used tools. We used TeraVR to recheck the reconstruction of neurons with very complex morphology, such as the cortico-cortical neurons, initially generated by annotators who had a lot of experience in using a popular reconstruction tool called Neurolucida (Neurolucida 360 or NL360*)*. Since NL360 does not have comparable capability to handle big data IO streaming, the annotators needed to load a portion of the imaging data at a time to reconstruct neurons, at a much slower pace. More importantly, upon rechecking in TeraVR we found imperfectness of these NL360-based reconstructions (Fig. 4a–c). The under-tracing of missing neurites was most notable, and the topology errors and over-tracing were common (Fig. 4a–d) even for the cells traced from overall clearly labeled brains. In some cases, more than 40% of neurites of a neuron were found to be missing (Fig. 4c, d and Supplementary Fig. 6a). Notably, a missing axonal branch at the proximal part of an axon was often seen, which indicated missing a long projection and the corresponding whole distal targeting axonal cluster (Fig. 4a, c). Also, annotators could choose to proceed along a wrong direction when a confusing branching region was encountered, which would lead to more severe reconstruction errors (Supplementary Fig. 6). These indicate the limitation of conventional tools for accurately observing neuronal structures in certain special situations such as dense neurites, axonal collaterals in dendrosomatic regions, where signals become obscure (e.g., long axonal collaterals extending along pia, Fig. 4c). Detailed examination of the ending points of three failure cases of NL360 (Supplementary Fig. 7) shows that the conventional 2-D annotation of single *z*-planes is not only too time-consuming but also fails to convey sufficient information to infer the continuity of neurites that is intuitively visible in TeraVR. The conventional 3-D maximum intensity projection method also fails for the cases because of weak signals and strong occlusion (Supplementary Fig. 7). This observed limitation is common for the non-VR approaches, such as Vaa3D-TeraFly and Neurolucida, compared to TeraVR. A careful examination of 17 complex neurons from three whole brains indicated that TeraVR extended 10–103% of the overall lengths of reconstructions from these neurons (Supplementary Table 1). We also carefully examined several other VR software packages and did not find any one that had comparable functions as TeraVR (Supplementary Tables 2 and 3).

### Collaborative annotation of TeraVR

**Fig. 5:**
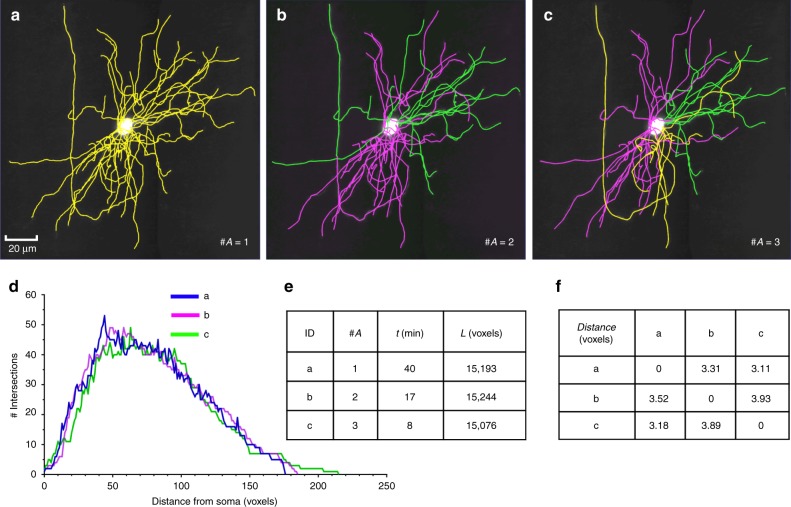
Results produced using the collaboration mode of TeraVR. **a**–**c** Reconstructions done by different numbers of collaborating annotators; different colors of neurites indicate parts done by different annotators; #A: number of annotators. **d** Sholl analysis of three reconstructions in **a**–**c**. **e** Summary of the number of annotators, reconstruction time, and the total length of reconstructions in **a**–**c**. **f** The pairwise spatial distance of reconstructions in **a**–**c**

In contrast to 2-D display devices in front of which multiple people may view the same visualization simultaneously, currently one 3-D VR headset can only be worn by one person at a time; therefore, an annotator may not communicate easily with others once this person is working in the VR environment. To overcome this limitation, in TeraVR we developed a collaboration mode with which multiple users can join the same session to reconstruct the same neuron at the same time, similar to the co-editing feature of Google Docs. Specifically, in TeraVR we implemented a cloud-based server-client infrastructure, with which the annotation data of individual annotators are streamed to the server in real time and merged with the data produced by other collaborating annotators. Users are able to see all annotations produced by others in real time and perform certain further annotations. We assembled a geographically remote team of annotators in Nanjing (China), Shanghai (China), and Seattle (USA) to use this collaboration mode to simultaneously reconstruct complicated 3-D neuron morphology from the whole-brain imaging dataset (Figs. 1d and 5). Three annotators, each from a different city, were able to co-reconstruct in real-time dendritic and axonal structures around the soma of a neuron (Fig. 5a–c) with only 20% of time compared to one single annotator (Fig. 5e). A Sholl analysis^21^ indicated that the TeraVR reconstructions produced by different combinations of annotators had consistent topology (Fig. 5d). A length analysis indicated that the difference of neuron lengths generated by such combinations of annotators was also small, at only 0.77% of the average total length of the reconstruction (Fig. 5e). A spatial distance analysis indicated that the average lateral apartness of these reconstructions was about 3.5 voxels, which was 0.05% of the longitudinal span of the neuron (Fig. 5f). This study indicates the power of TeraVR’s collaborative approach for remote annotation.

### Artificial intelligence enhanced TeraVR

We developed TeraVR as an open system, which can be augmented by a number of other programs without compromising its modularity. In particular, we enhanced TeraVR using several artificial intelligence (AI) techniques to further improve the efficiency of annotators. First, for the imaging data, we trained a deep-learning model, U-Net^22^, based on high-quality reconstructions produced using TeraVR; then in TeraVR we allowed a user to quickly invoke the trained U-Net to separate neurite signal from background (Supplementary Fig. 8a, b). We streamed the U-Net-filtered images in real time to TeraVR as an option that a user could choose. This U-Net model could also be iteratively refined based on user’s feedback; thus, it could be adapted to different brain images when needed. Second, for neuron reconstructions, in TeraVR we implemented a data-filtering model to detect various outlier structures, such as branches that had sharp turns (e.g., turning angle >90° or 135° or other user-specified values), and then generate alerts to allow users to immediately focus on the structures that might be traced with errors (Supplementary Fig. 8c, d).

## Discussion

TeraVR offers an immersive, intuitive, and realistic experience for exploring brain imaging data, similar to the mixed reality visualization shown in Fig. 1b and Supplementary Movie 12, where real and virtual contents were synthetically put together to demonstrate the user experience of TeraVR. While VR has not been widely used in biology, it is useful for biological problems, especially due to the intrinsic multi-dimensional nature of many biological datasets, and has the potential to be integrated as the next standard protocol. TeraVR is among the first demonstration of such utility with great potential. While immersive VR visualization of biological surface objects and sometimes also imaging data were shown in applications such as biological education and data analyses (Supplementary Table 2), there is little existing work on developing open-source VR software packages for very complicated and teravoxel-scale imaging datasets such as the whole-brain imagery as we have introduced here. We expect that TeraVR can also be used to analyze other massive-scale datasets, especially those produced using fast or high-resolution microscopy methods, such as the light-sheet microscopy^23–25^, expansion microscopy^26^, and recent nanoscale lattice microscopy^10^.

We chose to focus on applying TeraVR to the whole-brain single-neuron reconstruction challenge for two major reasons. First, currently no other alternative tools are able to reconstruct the fine, distal arborizations of neurons unambiguously in this way. Second, there has been little previous work on streamlining the large-scale data production of the complete single-neuron morphology at high precision and also at whole-brain scale. TeraVR has been a crucial tool to help several teams reconstruct precisely hundreds of full morphologies, with various image qualities, not only for single neurons but also for multiple densely packed neurons in very noisy images. These reconstructions have been released to the public databases, for example, NeuroMorpho.Org and the BRAIN Initiative Cell Census Network initiative (see Data availability).

Two additional aspects of TeraVR make this software package unique: the collaboration mode and the integration of the AI methods. TeraVR users can readily work together remotely and curate each other’s reconstructions. Such real-time ensemble annotation greatly improves the consistency, robustness, accuracy, speed, and actual fun of neuron reconstruction. With the further help of machine learning-based data analysis modules in both image and reconstruction domains, TeraVR will allow effective crowdsourcing and production of large-scale gold-standard reconstructions, which in addition to its inherent value will further help the automation of neuron reconstruction and systematic studies of neuron morphometry.

Integration of AI components in TeraVR can be implemented in a number of ways in addition to those examples shown in Results. A straightforward approach will be bundling existing intelligent tracing algorithms, such as the deep-learning-based DeepNeuron package^27^ or the reinforcement learning-based SmartTracing method^28^, in TeraVR to accelerate neuron reconstruction. AI could also be used in the TeraVR-based reconstruction workflow to check and ensure the integrity of the data repeatedly, using either cross-validation or even a generative adversarial networks model^29^. Since obviously TeraVR is useful not only for neuron reconstruction but also for a wide range of image datasets, especially in the context of Big Data, the integration of AI could also include artificial scene modeling for the virtual 3-D environment, AI-based data fetching for even faster data I/O, and so on. These technologies could extend the applications of TeraVR well beyond neuroscience to other domains like education, gaming, and medical applications (e.g., telesurgery).

## Methods

### Data preparation

*Tnnt1-IRES2-CreERT2;Ai82;Ai140* (brain ID nos. 17302 and 17545), *Gnb4-IRES2-CreERT2;Ai139* (no. 236174), and *Plxnd1-CreER;Ai82;Ai140* (no. 17300) mice were used in fMOST^30^ imaging to produce raw image stacks, which were further converted into the TeraFly format using Vaa3D’s module TeraConverter. All experiments related to the use of mice followed NIH guidelines, and received approval from the Institutional Animal Care and Use Committee.

### TeraVR visualization

TeraVR provides an immersive VR environment and true 3-D experience for interactive neuronal image visualization and annotation. A VR device, for example, the HTC Vive (https://www.vive.com/us/), typically has a wearable headset (also known as head-mounted device) with two independent monitors. The left monitor is exclusively viewed by the left eye, and the right monitor by the right eye. TeraVR produces and feeds two slightly different rendering streams for left and right monitors, which are viewed by the user simultaneously to create a realistic stereo visualization. We used the ray-casting technique to render neuronal volume images. To allow the user to observe the data inside the image volume, TeraVR adds a clipping plane orthogonal to the view direction to the typically used cube-model texture mapping to form a closed surface.

### Collaboration mode

TeraVR allows multiple annotators to work collaboratively during reconstruction. To enable the collaboration mode, a collaboration server is deployed on the cloud or in the intranet. The server receives messages from each connected annotator, and broadcasts to the others. An annotator joins a collaboration session by specifying the username and the IP/port of the collaboration server. Once connected, the annotator’s real-time working location in an image will be represented by an avatar, which is visible to all the other annotators. The annotator is also assigned a unique color, which is used as both the avatar’s color and the annotation’s color. When the annotator edits the reconstruction, for example, adding/deleting a neurite or a marker, the operation is converted to a globally understandable command, which is sent to the server. The server maintains a queue of commands and dispatches them in sequence to all the connected annotators. In this way, the reconstruction result is synchronized among all the annotators. It needs to be noted that as participating annotators might have different levels of knowledge of the data when they conducted the multi-party collaboration in Fig. 5b, c, there could be fluctuation of time reduction shown in Fig. 5e: in some situations there would be extra time-saving, while at different times there could be a slowdown to resolve conflicts.

### Mixed reality video making

To generate a mixed reality demonstration (Fig. 1b) that shows how TeraVR works, we first setup a physical camera to capture the movement of the annotator. A green screen was used to help remove the background. Meanwhile, an additional virtual camera was placed at the location of the physical camera (rather than being mounted on the VR headset) to generate a rendering stream of TeraVR from a third-person view. Importantly, the physical and the virtual cameras had exactly the same settings, including position, orientation, focus, and so, so that the real video stream was directly superimposed over the virtual one. These two cameras were started after TeraVR was launched. The mixed reality video was produced by synthesizing these two video streams.

### Profiling the image quality of a neuron

To evaluate how hard to reconstruct a neuron, we profiled the underlying image quality for a neuron. We first decomposed a neuron structure into a set of segments, each being bounded by a pair of critical points (branch points, terminal points, or the soma). The foreground (*F*), background (*B*), and critical background (*B*_crt_) were extracted for each segment: *F* was defined as the area enclosed within the radius of reconstructed neurite segment, *B* was defined as the bounding box of the segment excluding *F*, and *B*_crt_ was defined the 20% brightest voxels within *B*. We then calculated the SNR for a neurite segment as ${\mathrm{SNR}}= \frac{{\bar F}}{{\bar B_{{\mathrm{crt}}} + \varepsilon }}$, where *ε* is a small positive number and $\bar F$ and $\bar B_{{\mathrm{crt}}}$ were the average intensities for the image voxels in foreground and critical background, respectively. Four SNR ranges were defined based on annotators’ consensus opinions: very low for SNR ∈ (−∞, 1.0] (the neurite signal was either very weak or very noisy), low for SNR ∈ (1.0, 1.2] (the signal was still in low quality), mid for SNR ∈ (1.2, 1.4], and high for SNR values ∈ (1.4, ∞) (strong signals, which are clear and easy to trace). The overall image SNR of a neuron was calculated as the segment-wise average SNR weighted by the length of each segment.

### Computer configuration

TeraVR was implemented and evaluated on computers with Intel Core i7-7700 CPU @ 3.60 GHz, 64 GB memory, NVIDIA GeForce GTX 1070 GPU, Windows 10 64-bit edition, and HTC Vive as the VR device.

### Compatibility

TeraVR can be used to explore multi-dimensional, multi-channel image data, as long as the data format is supported by Vaa3D. For very large-scale images (e.g., those with 100+ billion voxels), it is recommended to organize the data in the Vaa3D-TeraFly format for smooth performance.

### Reporting summary

Further information on research design is available in the Nature Research Reporting Summary linked to this article.

## Supplementary information

Supplementary Information

Description of Additional Supplementary Files

Supplementary Movie 1

Supplementary Movie 2

Supplementary Movie 3

Supplementary Movie 4

Supplementary Movie 5

Supplementary Movie 6

Supplementary Movie 7

Supplementary Movie 8

Supplementary Movie 9

Supplementary Movie 10

Supplementary Movie 11

Supplementary Movie 12

Reporting Summary

## Data Availability

Whole-brain test imaging data is available upon request due to their large sizes. The neuron reconstructions released are deposited to public databases, such as NeuroMorpho at http://neuromorpho.org/dableFiles/allen%20cell%20types/released_annotations.tar.gz.

## Code availability

TeraVR is released Open Source, as part of Vaa3D (e.g. https://github.com/Vaa3D/release/releases/download/v3.597/vaa3d_teravr.zip). A user guide for TeraVR is provided in Supplementary Note 1.
