# Supplementary material for: TeraVR empowers precise reconstruction of complete 3-D neuronal morphology in the whole brain
Source: Nat Commun. 2019 Aug 2;10:3474. doi: 10.1038/s41467-019-11443-y (PMC6677772; doi:10.1038/s41467-019-11443-y)
Supplement: Supplementary file 1 — Supplementary Information [file 41467_2019_11443_MOESM1_ESM.pdf]

Supplementary Information

"TeraVR Empowers Precise Reconstruction of Complete 3-D Neuronal Morphology in the Whole Brain", Wang *et al*.

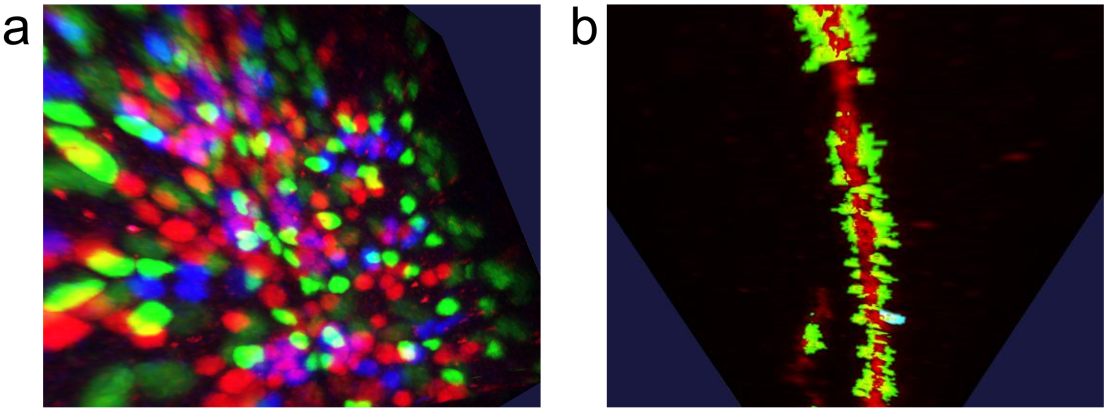

Supplementary Figure 1. Visualization of images of multiple channels using TeraVR. **a** A group of cells labeled in red, green, and blue colors. **b** An image stack in which the dendritic neurites are visualized in red color, and the spines are visualized in green color.

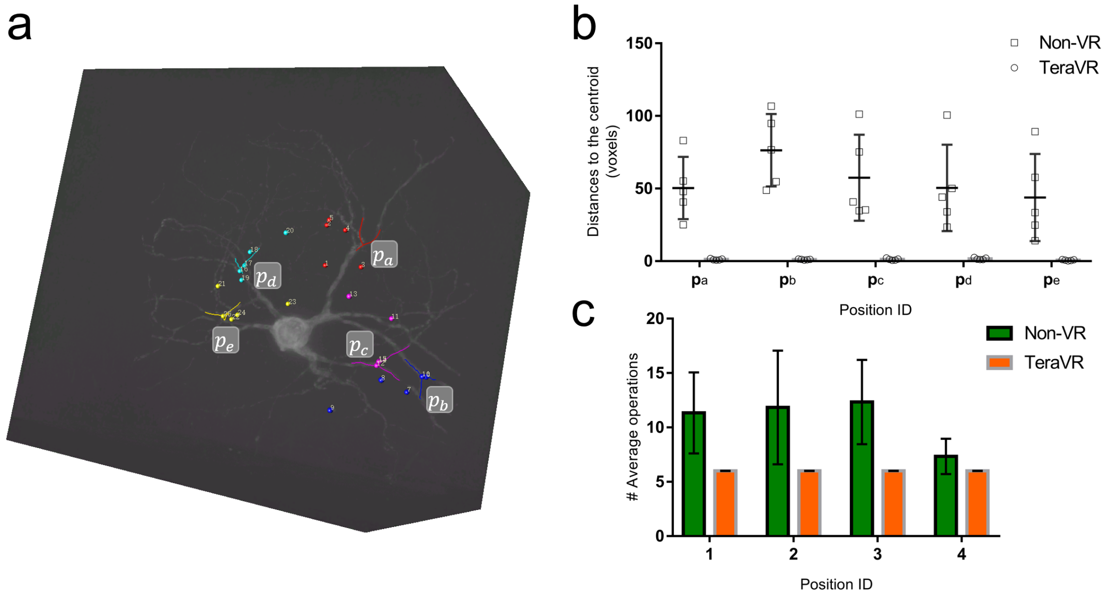

Supplementary Figure 2. Accurate pinpointing in TeraVR. **a** A local image volume that contains a dendritic tree. 5 bifurcations are highlighted using Y-shaped structures of different colors. 5 attempts for adding a marker at each of the bifurcations are made using TeraVR and non-VR approach, respectively. The displayed markers are the according attempts to pick up the bifurcations using non-VR approach. **b** For each group of attempts, a geometric centroid is calculated. The plot shows the distance to the centroid; error bars: S.D. **c** A plot of number of operations needed in order to go to the highest resolution of a ROI from the lowest resolution. 4 different locations in a whole-brain image were used for testing. TeraVR has stable performance and requires only the fewest number of operations (i.e. 6 zoom-in operations to precisely arrive at the level-7 resolution of the ROI from the level-1 resolution). The non-VR approach lacks enough accuracy for pinpointing and thus needs more operation to accomplish the task; error bars: S.D.

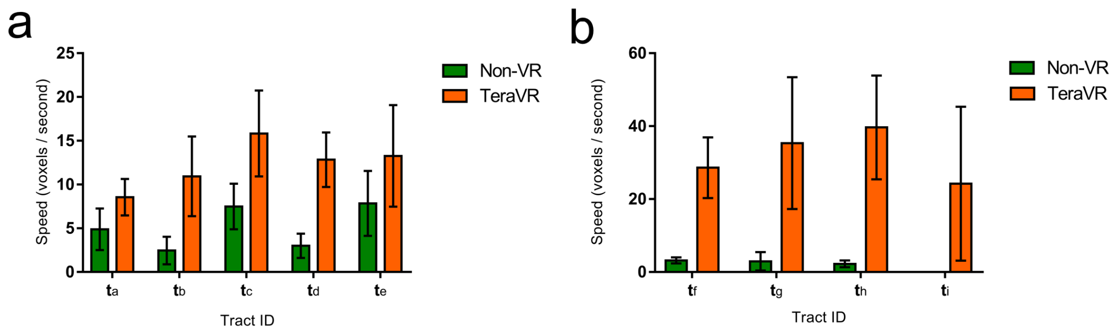

Supplementary Figure 3. The reconstruction speed of TeraVR vs non-VR. **a** The reconstruction speed of five tracts in **Fig. 2a**. **b** The reconstruction speed of four tracts in **Fig. 2c**, where the speed for tract t_i using non-VR approach is zero due to the lack of succeeded attempts; error bars: S.D.

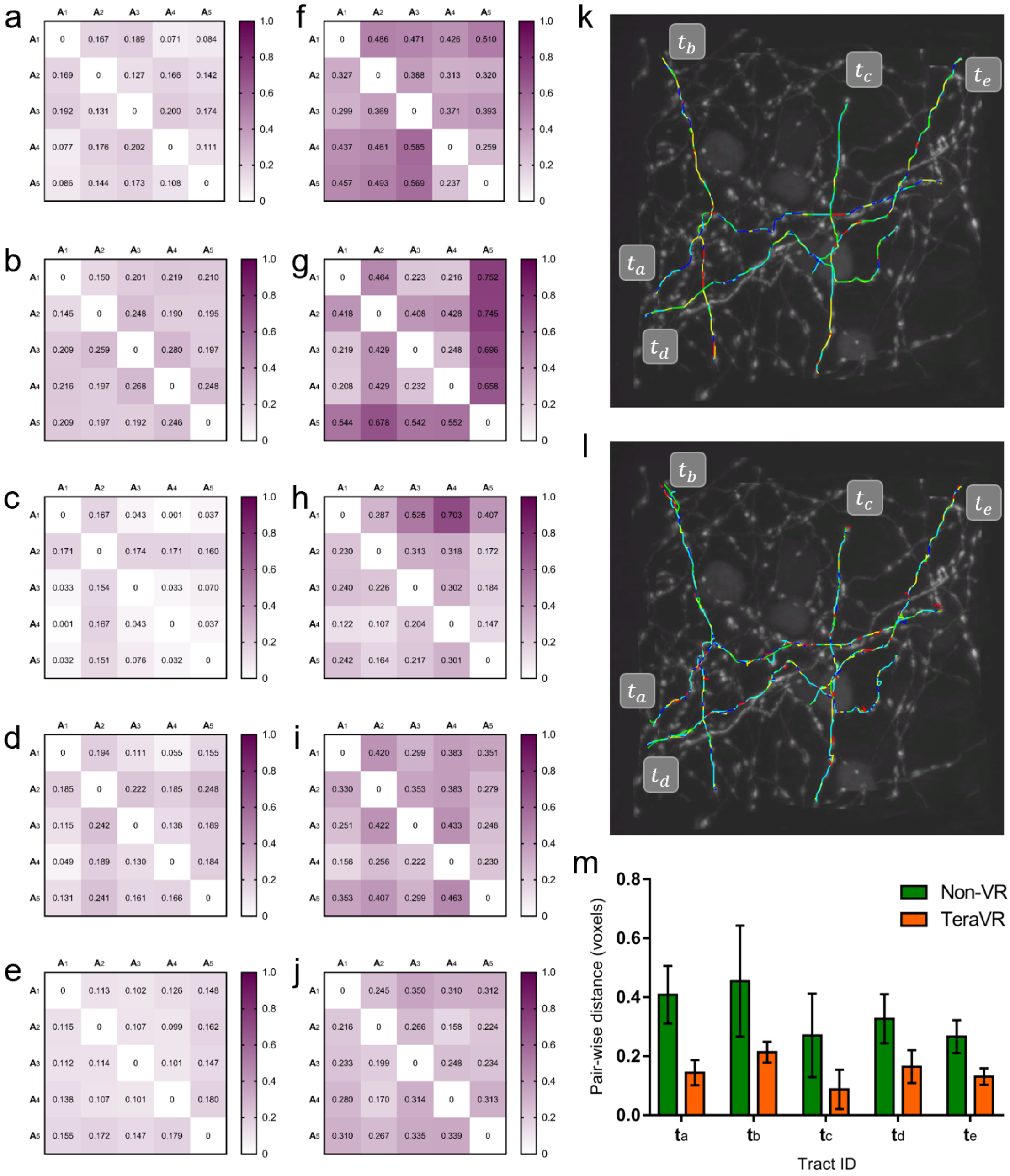

Supplementary Figure 4. TeraVR consistency. **a-e** The spatial distance matrices of independently generated TeraVR reconstructions for tracts t_a, t_b, t_c, t_d, and t_e in **Fig. 2a** by 5 annotators A_1~A_5 independently. The smaller the spatial distance, the more consistent (similar) these reconstructions. Refer to Peng, H. et al., 2010[1] for the calculation of the spatial distance. **f-j** The spatial distance matrices of independently generated non-VR reconstructions for tracts t_a, t_b, t_c, t_d, and t_e in **Fig.2a**, respectively. **k** Overlaid TeraVR reconstructions (each reconstruction in a different color). **l** Overlaid non-VR reconstructions (each reconstruction in a different color). **m** The average pair-wise reconstruction distances for each tract; error bars: S.D.

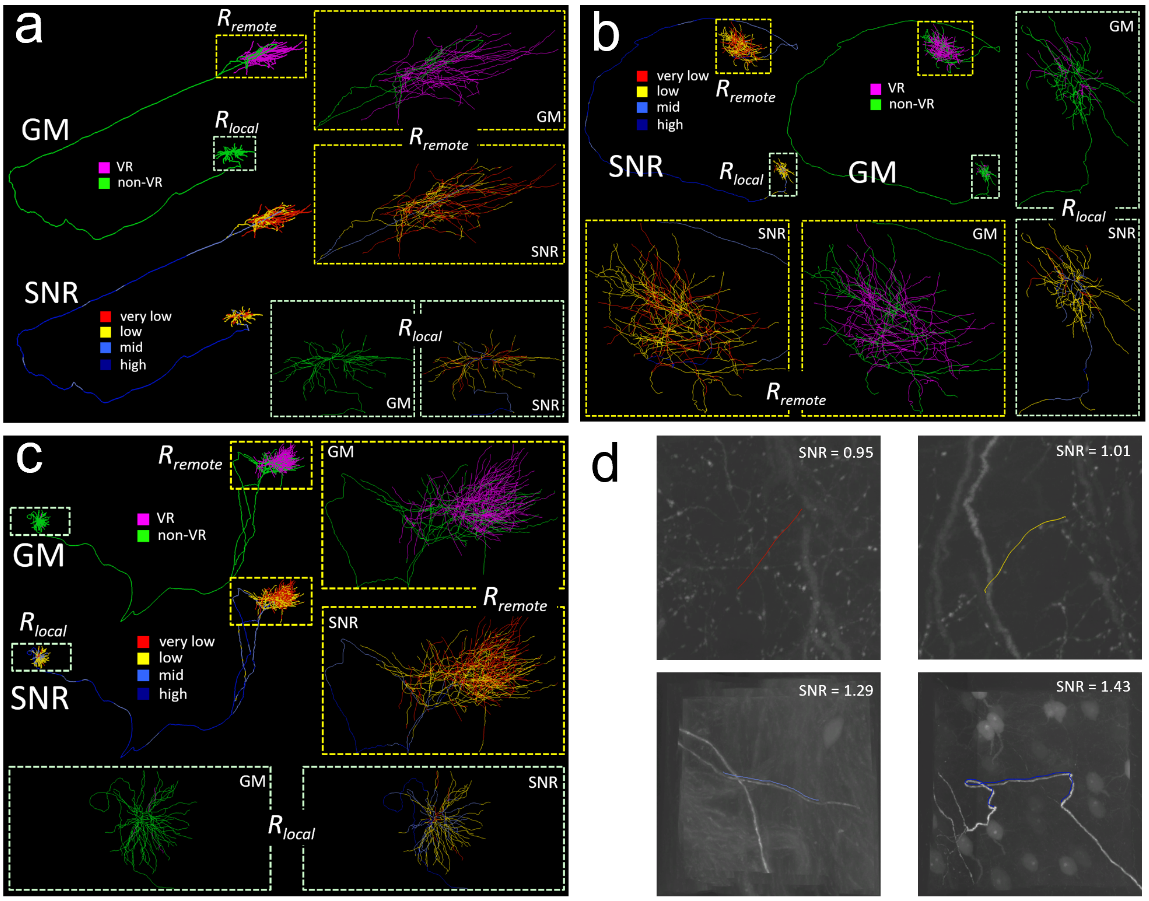

Supplementary Figure 5. More complete reconstructions of neurons at whole brain scale using TeraVR. **a-c** 3 more neurons reconstructed using TeraVR. Refer to **Fig. 3** for the meaning of the color-coding. **d** The illustrations of image regions with various SNRs. The neurites are given a slight offset for clarity.

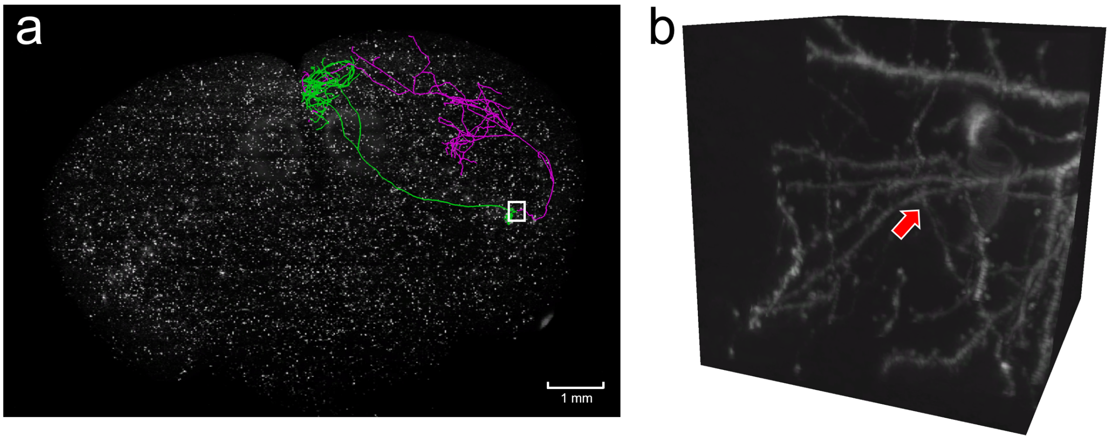

Supplementary Figure 6. TeraVR helps picking up a major missing cluster in neuron-reconstruction produced first with Neurolucida. **a** A neuron first produced with Neurolucida (green) and then corrected by TeraVR (magenta), overlaid on the whole-mouse brain imaging data. The image quality is challenging in the region specified by the white box, leading to a large size of incorrectly-traced arbor in the Neurolucida reconstruction that was later identified and deleted using TeraVR (the deleted part of the Neurolucida reconstruction is not displayed here). **b** Full-resolution imaging data corresponding the to the white box in **a**. The red arrow points to the critical position that corresponds to the reconstruction error resulting in a major missing cluster.

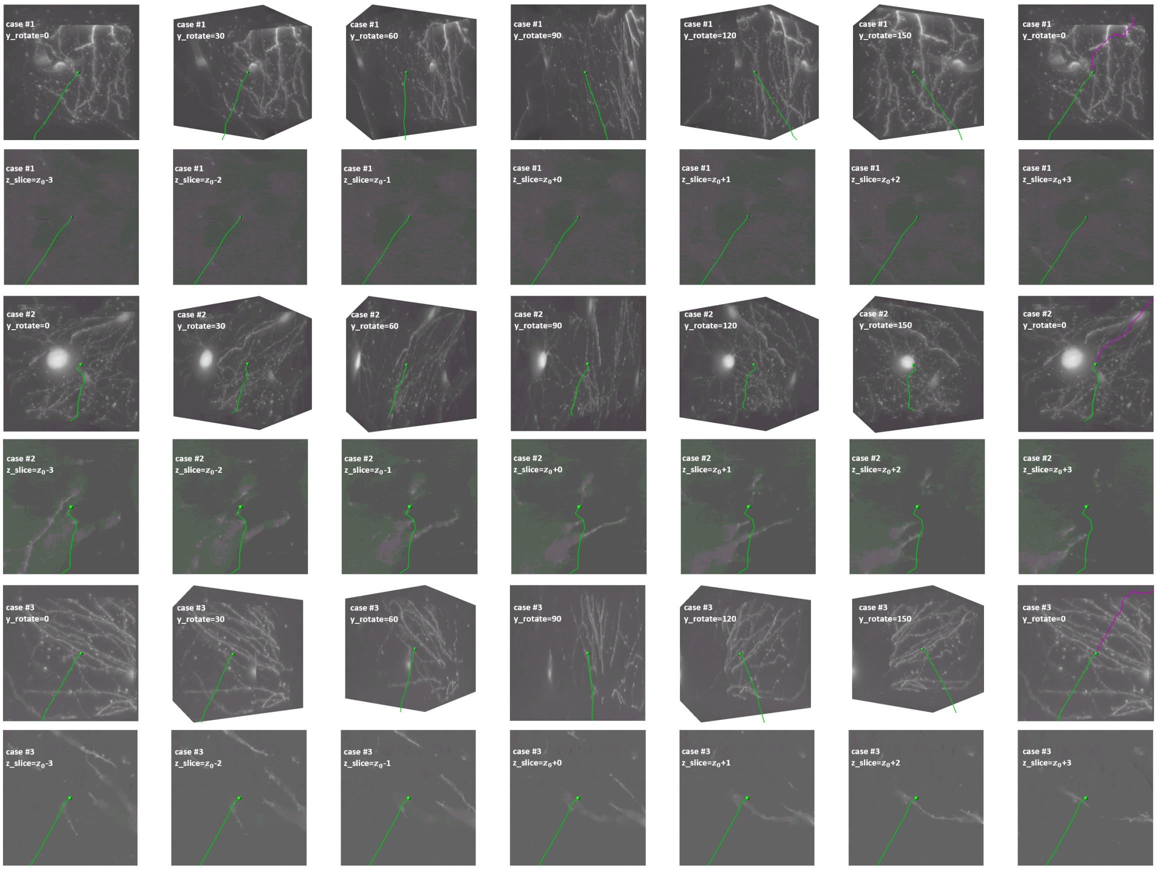

Supplementary Figure 7. Detailed examination of three failure cases of NL360, compared to TeraVR. For each case, the respective wrong ending location of the NL360 tracing (green tract) is checked carefully in two ways: 3-D maximum intensity projection (MIP) and single 2-D z-sections. For MIP, 6 rotation angles around the y-axis, centered at the respective ending location (x_0,y_0,z_0), are shown (rows 1, 3, and 5 in this figure). For single 2-D z-sections, 7 continuous z-sections around the respective ending location (x_0,y_0,z_0) are shown (rows 2, 4, and 6 in this figure). For reference, the TeraVR-edited results (magenta in addition of green) are also shown in the top-right corners for these cases (column 7 of rows 1, 3, and 5).

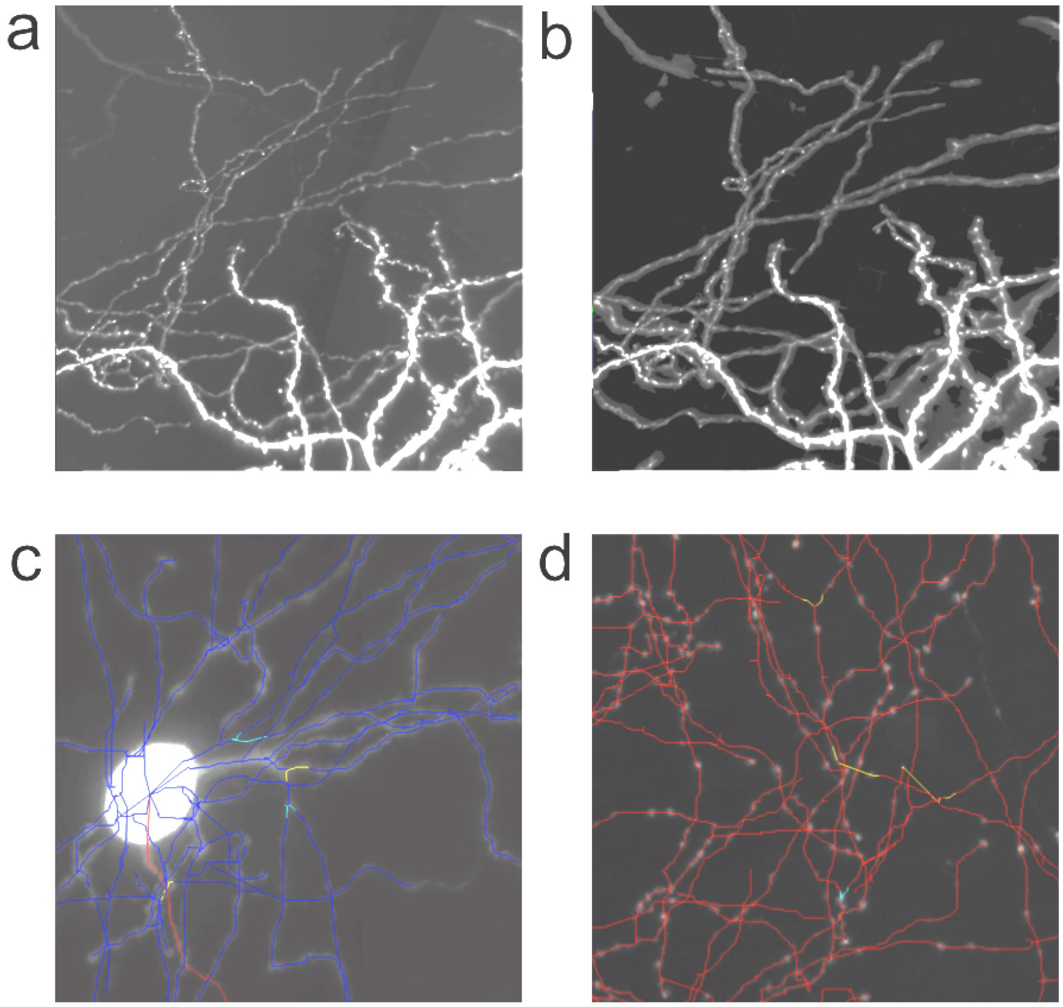

Supplementary Figure 8. Enhancing TeraVR using several AI modules. **a** The original image visualized in TeraVR. **b** The U-Net optimized image visualized in TeraVR. **c** A partial dendritic tree, where bifurcations with abnormal angles are highlighted (non-blue/red colors). **d** A terminal axon arbor, where bifurcations with abnormal angles are highlighted (non-red colors). For all subfigures, brightness +40%, contrast -40% for more visibility.

Supplementary Table 1. Statistics for 17 neuron-reconstructions (from three brains) that were first reconstructed using Neurolucida and then corrected using TeraVR.

Brain ID	Neuron ID	Total length (mm)	TeraVR length (mm)	Non-VR length (mm)	% increased
236174	04229-04328-X13663-Y8589	67.6354	34.3252	33.3102	103.0471%
17300	5969-X27278-Y20820	63.5335	26.2606	37.2729	70.4549%
17300	03514-3525-X19676-Y45282	51.6424	16.1144	35.528	45.3569%
236174	03329-03428-X13938-Y26099	74.4109	22.4904	51.9205	43.3170%
17545	05574-X24399-Y33944	48.878	12.954	35.924	36.0595%
236174	03536-03545-X15159-Y25525	93.6728	19.3034	74.3693	25.9561%
17545	05689-X21900-Y16152	21.3688	3.85913	17.5096	22.0401%
17545	06151-X24259-Y36270	21.5348	3.85515	17.6797	21.8055%
236174	03529-03628-X12805-Y10541	106.858	18.2958	88.5619	20.6588%
236174	03001-03008-X12887 Y24248	115.714	18.3905	97.3237	18.8962%
17545	06070-X20183-Y17777	27.1727	4.217	22.9557	18.3702%
17545	06034-X23713-Y35681	42.3159	6.34982	35.966	17.6551%
17545	05534-X20427-Y33851	54.3758	7.94209	46.4337	17.1042%
17545	05996-X19743-Y18066	30.3852	4.07729	26.3079	15.4983%
17300	03426-X20339-Y44872	65.7337	8.45904	57.2747	14.7692%
236174	03429-03528-X12632-Y10625	97.0815	11.2848	85.7968	13.1529%
236174	03447-03459 X12562 Y10626	73.5985	7.13707	66.4614	10.7387%

Supplementary Table 2. VR software for scientific visualization. *syGlass has preliminary support for neuron reconstruction by placing consecutive nodes in the space to represent neurites, which is not practical and efficient for use in complete neuron reconstruction from whole-brain data. Ref_1: https://www.arivis.com/en/imaging-science/arivis-inviewr . Ref_2: https://www.syglass.io/ .

	arivis InViewR	syGlass	ConfocalVR	VRNT	TeraVR
VR visualization of 3D images	Yes	Yes	Yes	Yes	Yes
VR neuron reconstruction	No	Preliminary*	No	Yes	Yes
Reported application to whole brains	No	No	No	No	Yes
License	Commercial	Commercial	Free only for nonprofit	Free	Free and Open Source
Reference/link	Ref_1	Ref_2	2	3	This article

Supplementary Table 3. A comparison between TeraVR and VRNT regarding the data management and compatibility, functions for reconstruction, and visualization and navigation. **Bold**: critical features.

	Features	VRNT	TeraVR
Data management and compatibility	Support for imaging data	Yes	Yes
	**Size of tested imaging data (voxels)**	300 megavoxels	10~30 teravoxels
	Support for multi-channel imaging data	No	Yes
	Direct opening of morphology data	No	Yes
Functions for reconstruction	Adding or deleting tracts	Yes	Yes
	Modifying the fine geometry of a tract	No	Yes. A number of functions such as subdivision and dragging are provided.
	Undo	Yes	Yes
	Redo	No	Yes
	**Semi-automatic reconstruction**	No. Reconstruction results might not well align with the signal.	Yes. Virtual finger makes neurites align with signals.
	**Collaborative reconstruction**	No	Yes
	**Support for artificial intelligence**	No	Yes
Visualization and navigation	**Visualization mode**	Single. VR mode only.	Dual. Allow convenient switch between VR and non-VR mode.
	**Multiresolution display**	No. Thus not applicable for whole-brain applications w/o redevelopment.	Yes. It is helpful when working on whole-brain imaging data.
	**Good visibility of complex, weak and/or noisy regions**	No. Visibility is not good in many challenging cases.	Yes
	Contrast adjustment	No	Yes
	Hide reconstructions	No	Yes
	Translating	Yes	Yes
	Rotation	No	Yes
	Scaling	No	Yes. It could be useful for anisotropic imaging data.

Supplementary Note 1. User Guide of TeraVR

1.1 Installation and environment setup

TeraVR is released Open Source, as part of Vaa3D (https://github.com/Vaa3D/release). References and protocols for installing and using Vaa3D can be found at Peng, H. *et al.*, 2014[4]. While Vaa3D can be used directly on a common workstation or laptop, using TeraVR requires a virtual reality environment to be properly deployed. Currently, TeraVR is compatible with off-the-shelf VR equipment such as HTC Vive and Vive Pro. The user can go to https://www.vive.com/setup/ to download the software for Vive and follow the on-screen instructions to install it. During installation, the user will also be guided to set up the lighthouses, connect the headset to the computer or laptop, pair the Vive controllers, and initialize the virtual environment. A detailed instruction on Vive installation can be found at https://support.steampowered.com/steamvr/HTC_Vive/ .

Sometimes, there might be needs for deploying multiple TeraVR systems in a same workspace. In that case, each computer running TeraVR needs to be independently equipped with a Vive headset and two Vive controllers. However, they can conveniently share a same pair of lighthouses for movement tracking (**Supplementary Fig. 9**).

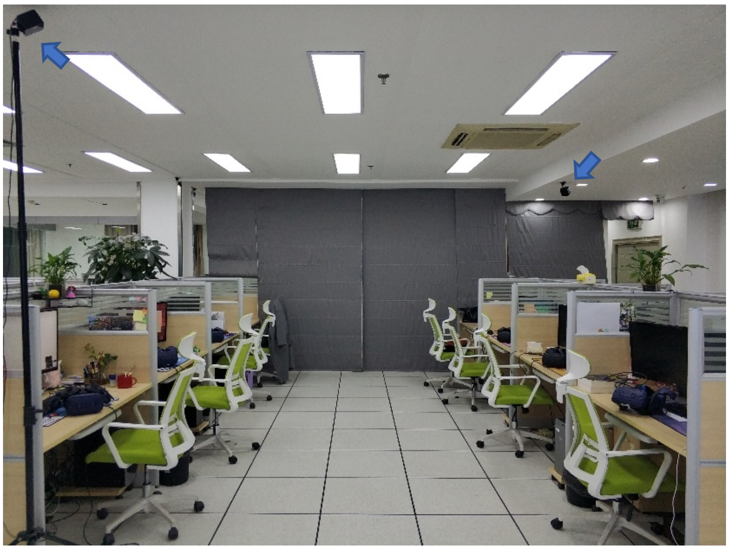

Supplementary Figure 9. A setup of multiple TeraVR systems in a shared workspace. In the scene, there are 6 sets of VR/TeraVR systems, all of which share a single pair of lighthouses (pointed by arrows).

1.2 User Interface

1.2.1 Entering and exiting TeraVR

First, the user needs to start Vaa3D software, load an image volume, a surface object, or a whole-brain data, power on the Vive device, and also launch the Steam VR software. Next, TeraVR can be launched from either a normal 3-D viewer or a TeraFly 3-D viewer of Vaa3D by clicking the "**See in VR**" button, as shown in **Supplementary Fig. 10**. After that, the user

can put on the VR headset to start using TeraVR, and all the contents previously in the 3-D viewer are now observable in TeraVR. In addition, a **companion window** that shows the replicated view in the headset is displayed on computer monitor (**Supplementary Fig. 11**), in order for other users to know what is currently being seen in VR.

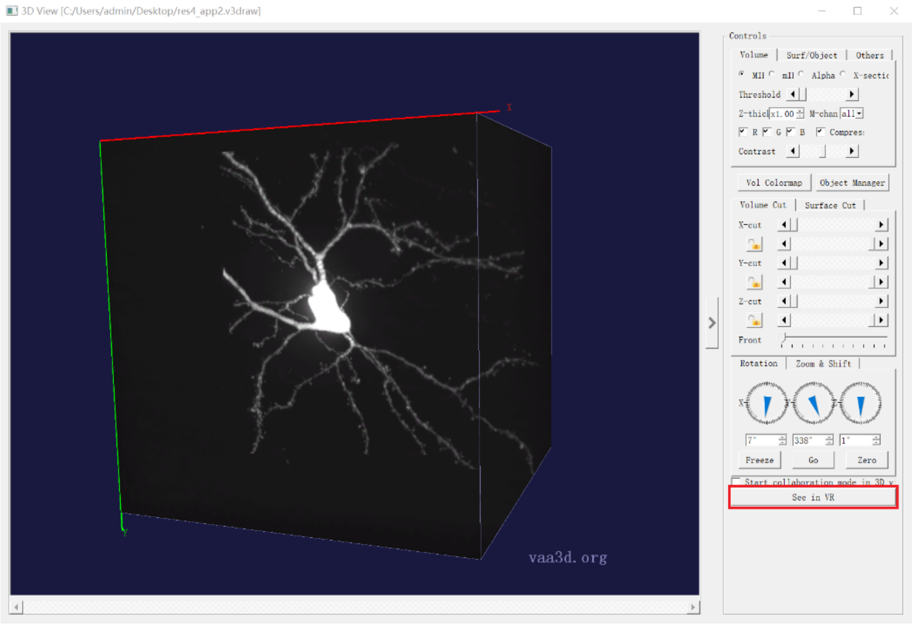

a

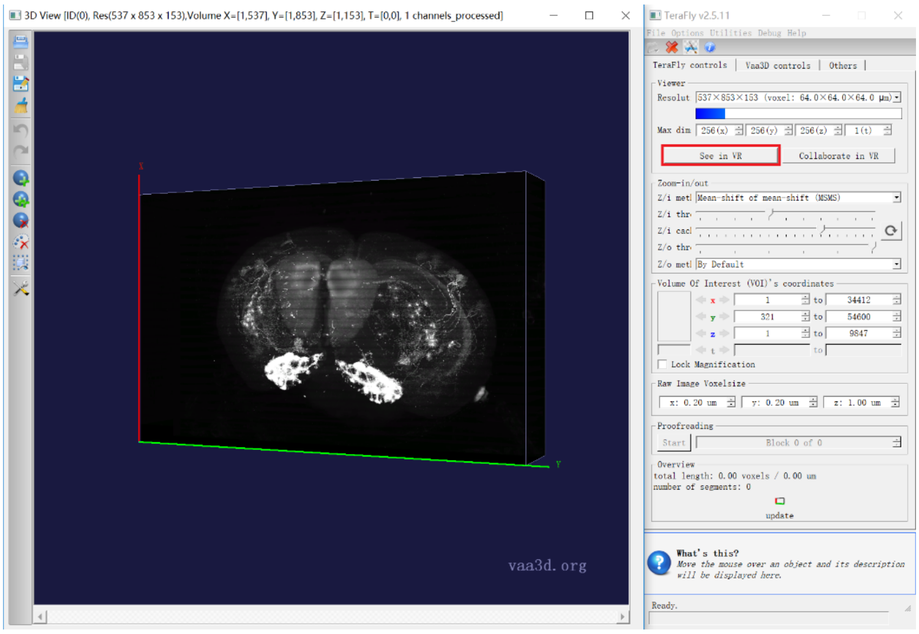

b

Supplementary Figure 10. Entering TeraVR from Vaa3D. **a** A normal 3-D viewer. **b** A TeraFly 3-D viewer. The “See in VR” button is highlighted in both (a) and (b).

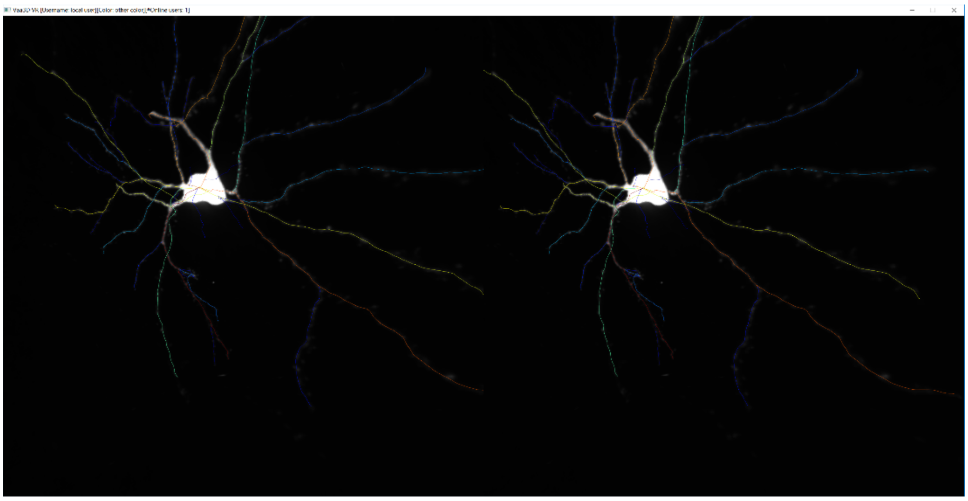

Supplementary Figure 11. The companion window showing left-eye and right-eye views of TeraVR.

To exit TeraVR, the user can either press the exit button on the setting controller (see **Supplementary Fig. 12a**) or press the ESC button on the keyboard. After exiting, all the latest annotation results in TeraVR are brought back to the 3-D viewer, where the user can continue to edit.

1.2.2 User interactions in TeraVR

User interactions in TeraVR is mainly carried out with the two handheld controllers. These two controllers can be used either individually or simultaneously. Two corresponding virtual models of the controllers are shown in TeraVR, which have synchronized locations with the physical devices. The **annotation controller (AC)** is mainly used for annotation purpose (**Supplementary Fig. 12a**), and the **setting controller (SC)** is mainly used to adjust various settings (**Supplementary Fig. 12b**). Each controller has a touchpad and a trigger button (**Supplementary Fig. 12 a-b**). Their functions will be described in detail in the following sections. Besides, AC has a **Menu** button 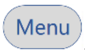

, and SC has a **Quit** button 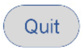

. The menu button is used to activate/deactivate the menu for various TeraVR functions and settings. When the menu button is pressed, the menu for major TeraVR functions (**Supplementary Fig. 12c**) appears on top of SC. Pressing the menu button again will hide the menu. In order to select a menu item, the user aims the red-colored shooting ray from AC at the desired menu item, and presses the trigger button of AC.

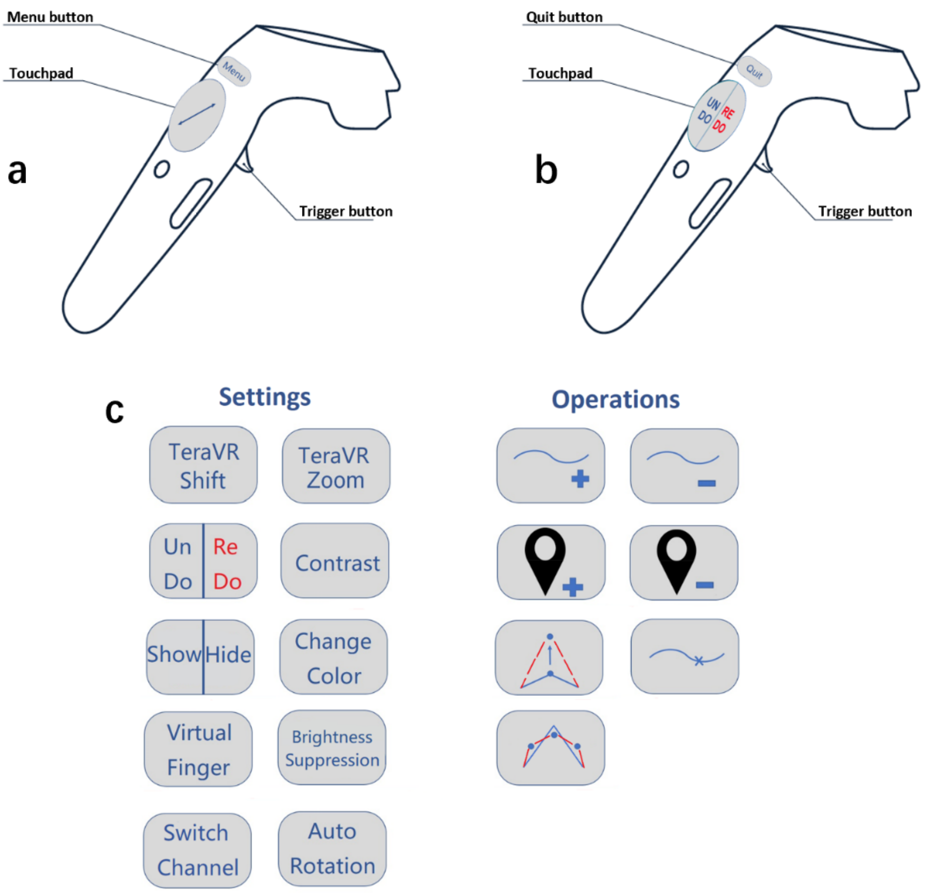

Supplementary Figure 12. The controllers and the menu interface in TeraVR. **a** Annotation controller. **b** Setting controller. **c** The TeraVR menu interface.

1.3 Visualization

TeraVR is based on stereo visualization and provides an intuitive tool for observing 3-D imaging data and 3-D objects. In TeraVR, real and virtual worlds are synchronized. When a user wearing the VR headset changes the viewing direction by turning the head or moving around, the contents being visualized are automatically updated according to the new viewing direction, which brings a very similar experience as the real world perception. Also, just like dealing with physical objects, the user can virtually hold 3-D imaging data and objects in hand and rotate around to adjust observation perspective. This can be done by holding the trigger button of SC and rotating the controller.

Current imaging techniques often generate anisotropic data, where the z resolution is not as high as the x and y resolutions. TeraVR supports non-uniform scaling of imaging data, and the user can stretch the data in the z direction by touching the 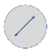 button on the touchpad of AC. If the upper half of the button is touched, the z direction keeps scaling up; if the bottom half is touched, the z direction keeps scaling down.

TeraVR supports hierarchical management and visualization of teravoxel-scale images. To zoom-in/zoom-out across different resolution levels, select the 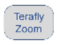

button from the menu,

and press the 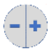 button on SC accordingly. To shift to an adjacent image volume of the same resolution level, select the 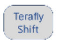 button from the menu, place SC at a desired position, and press the 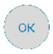 button on SC. Depending on the relative position between the SC and the center of the image volume, a corresponding adjacent image volume will be fetched and displayed. Refer to **Supplementary Movies 1 and 2** for further demonstration.

There are also several settings and functions related to visualization, such as adjusting contrast. Refer to 1.5 for details.

1.4 Annotation

TeraVR provides various tools for 3-D neuron morphology annotation. With TeraVR, the user works in a true 3-D environment and has full degrees of freedom while interacting with 3-D imaging data and objects. The user can precisely pinpoint a 3-D location in the virtual world in an intuitive way (e.g., by posing a controller to a desired location) and carry out various annotation tasks, such as tracing neurites, adding markers, modifying geometry, etc. This section describes the major annotation functions of TeraVR.

1.4.1 Adding a neurite

To add a neurite, the user opens the menu, selects the 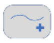 icon for the "Add neurite" function, and closes the menu after that. Next, the user finds a suitable viewing perspective, aims at the desired neurite signal with the sphere on AC, and traces the neurite by moving the sphere along it while holding the trigger button of AC. Refer to **Supplementary Movie 3** for further demonstration of adding neurites.

TeraVR provides two modes for tracing a neurite, i.e. the virtual finger (VF) mode and the non-virtual finger (non-VF) mode. With the VF mode, the drawn segment will automatically align with the signal in the imaging data to achieve high accuracy and consistency in tracing. With the non-VF mode, TeraVR will generate a free-form curve that strictly follow the moving path of AC. In general, we can use the VF mode to trace relatively strong signals and use the non-VF mode to trace extremely weak signals.

When the newly traced neurite is close enough to an existing one, TeraVR will automatically attach them together to form a connected neuronal tree. To differentiate structures that have different biological meanings, the user can assign different colors to each curve and marker. If the white color is used, the current neurite will adopt the color of the neurite which it connects to. For settings of the virtual finger mode and the tracing color, refer to Section 1.5 for instructions.

1.4.2 Deleting a neurite

To delete a neurite, the user selects the 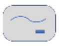 icon in the menu. After the function is selected, the user moves AC near a neurite to be deleted and presses the trigger once. TeraVR will find a nearest neurite around the AC and delete it. If there is no nearby neurite, none will be deleted. Refer to **Supplementary Movie 4** for further demonstration.

1.4.3 Adding a marker

Sometime the user needs to put markers at specify locations of interest. To do that in TeraVR, the user selects the 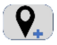 icon in the menu. Then, the user moves AC to the location of interest, aims with the sphere on AC, and presses trigger once. A marker can thus be added at that location in the currently chosen color. Refer to **Supplementary Movie 5** for further demonstration. To change color for adding markers, refer to Section 1.5.4.

1.4.4 Deleting a marker

To delete a marker, the user selects the 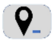 icon in the menu. Then, aim at the marker to be deleted with the sphere on AC, and press trigger once. Refer to **Supplementary Movie 5** for further demonstration.

1.4.5 Dragging a node on a neurite

Sometime, the user would like to fine tune the geometry of an existing neurite, in order to further improve the precision of reconstruction. TeraVR provides the node dragging function that allows one to modify the location of individual nodes on neurites. To do that, the user selects the 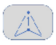 icon in the menu. After the function is selected, the user reaches a local region of a neurite with AC and holds the trigger button. A node on this neurite is thus locked by the AC. Then, without releasing the trigger, the user moves the node to a desired location and releases the trigger. The geometry of the neurite will then be modified accordingly. Refer to **Supplementary Movie 6** for further demonstration.

1.4.6 Splitting a neurite

To split a neurite into two smaller ones, the user selects the 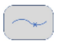 icon in the menu. Next, the user aims at the location to be split on the neurite with the sphere on AC and presses the trigger once. After that, the original neurite is broken into two neurites. The user can continue to edit these new smaller neurites, e.g. deleting one of them. Refer to **Supplementary Movie 6** for further demonstration.

1.4.7 Subdividing a neurite

Sometime, the number of nodes of a neurite is too few to precisely represent the exact signal in the imaging data. In such cases, the user can use the subdivision function to add more nodes to a neurite to increase its representation capability. First, select the 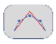 icon in the

menu. Then, use the sphere on AC to aim at the neurite where more nodes are desired. Next, press the trigger once. As a result, the subdivided neurite will now have more nodes which allows the user to modify the geometry in detail. Normally, there would be slight deformation on the neurite to indicated that the subdivision succeeded. Refer to **Supplementary Movie 6** for further demonstration.

1.5 Settings and tools

This section describes several settings and tools in TeraVR to facilitate neuron reconstruction. Most of the settings will be remembered even the user temporarily quits from TeraVR.

1.5.1 Undo / Redo

TeraVR supports undo and redo operations during neuron reconstruction. First, the user selects the icon, and the button appears on the touchpad of SC. To undo a previous operation, the user presses on the left half of the button; similarly, to redo a revoked operation, the user presses on the right half of the button. TeraVR supports 5 levels of undo and redo. Refer to **Supplementary Movie 4** for further demonstration.

1.5.2 Adjusting contrast

During neuron reconstruction, the strength of signal varies across the neuronal image, and the user usually needs to adjust the contrast of image from time to time, in order to have the best observation of the signal. In TeraVR, the user can first select the icon, and then use the button on the touchpad of SC to adjust contrast. Press left half of the button to decrease contrast level and the right half to increase contrast level. Press the button one or more times to obtain the ideal effect. Refer to **Supplementary Movie 7** for further demonstration.

1.5.3 Showing or hiding the annotation

In TeraVR, a user can choose to temporarily hide the annotation in order to inspect the imaging data more clearly, and to show the annotation again later to continue the reconstruction. To do that, the user selects the icon from the menu, and presses the or button on the touchpad of SC to toggle the show/hide status. Refer to **Supplementary Movie 8** for further demonstration.

1.5.4 Changing the color for tracing

The color for tracing is indicated by the sphere on AC. To change the tracing color, open the menu and select the icon. As a result, an button will appear on the touchpad of SC. While the user presses the button, the tracing color will be switched among a set of

colors. Once a color is set, all the neurites and markers added later will adopt that color. Refer to **Supplementary Movie 9** for further demonstration.

1.5.5 Toggling the virtual finger mode

To toggle between virtual finger mode and non-virtual finger mode, the user opens the menu can select the 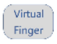

icon. Then, there will be a 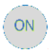

or 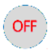

button on the touchpad to indicate the status of the VF mode. Press the touchpad to toggle the VF mode. Refer to **Supplementary Movie 3** for further demonstration.

1.5.6 Brightness suppression

Neuronal imaging data might have uneven distribution of signal strength even in a local region which could cause difficulties for reconstruction. For example, while the dendrites are relatively bright, the axonal neurites around soma area can be quite dim and thus difficult to observe even after adjusting contrast. TeraVR has the brightness suppression function, which can be used to suppress the bright signals and make the dim structure easier to observe. To do that, the user selects the 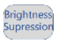

icon from the menu, and presses the 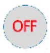

or 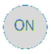

button on the touchpad of SC to enable or disable it. Refer to **Supplementary Movie 7** for further demonstration.

1.5.7 Switching channels of an image

TeraVR can be used for the visualization of both single-channel and multi-channel imaging data. For multi-channel data, TeraVR lets the user show all the channels simultaneously, or switch among individual channels. To switch channels, the user selects the 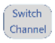

icon from the menu, and a 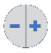

button will appear on the touchpad of SC. Press either "-" or "+" to switch among red/green/blue/all channels, in a forward or backward order. Refer to **Supplementary Movie 10** for further demonstration.

1.5.8 Automatic rotation

For 3-D imaging data, sometimes inspecting data from various angles is preferred in order to accurately understand the underlying structure. In TeraVR, the user can select the 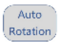

icon from the menu for the automatic rotation of 3-D data. To enable automatic rotation, the user presses the 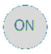

button on the touchpad of SC. Then, the data will start to rotate automatically using the position of SC as the rotation origin. The user can press the 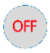

button to stop it. Refer to **Supplementary Movie 11** for further demonstration.

1.6 Collaboration mode

Reconstructing full morphology of neurons is a challenging task, and a collaboration mechanism can be helpful for both neuron reconstruction and proofreading. The collaboration

mode of TeraVR allows multiple users to work collaboratively on a same piece of data. These users can work together in a same workspace or be located worldwide.

To use the collaboration mode, first a cloud-based server needs to be launched to perform user management, synchronize reconstruction data, and coordinate actions among multiple users. The server program can be found at https://github.com/Vaa3D/vaa3d_tools/tree/master/hackathon/liqi/VR_Server.

The collaboration mode of TeraVR can be initiated both from normal 3-D viewers for regular image data and from TeraFly 3-D viewers for whole-brain image data. Take the latter case here as an example. In the TeraFly 3-D viewer (**Supplementary Fig. 10b**), the user clicks the "Collaborate in VR" button, and will be prompted to input server address, server port, and login name (**Supplementary Fig. 13**). After entering the above information, it will connect to the collaboration server, and start TeraVR with collaboration. Any other users that are working on the same whole-brain imaging data can join the collaboration with the same procedure. The number of users currently in collaboration is displayed on the title of the companion window.

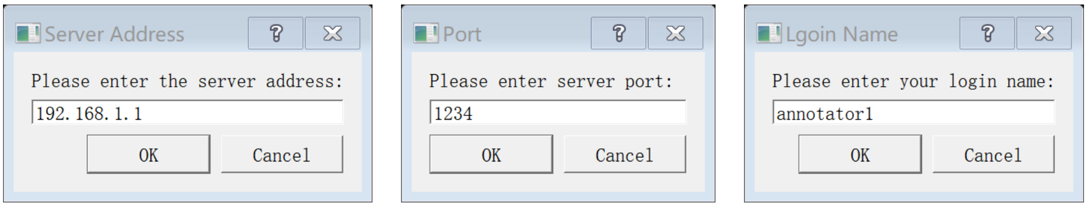

a **b** **c**

Supplementary Figure 13. Setting up connection to the server for collaborative reconstruction. **a** Input of IP address. **b** Input of port number. **c** Input of login name.

In the collaboration mode of TeraVR, an avatar for each user that represents the user's instant position is displayed, and its position is updated when the user moves. Therefore, a user is aware of where other people are working at. Also, each user is assigned with a unique color, which is used for both the avatar and the reconstructions done by the user.

Basically, annotation operations in collaboration mode do not differ too much with those in standalone mode. Only that during collaboration, reconstruction results are shared in real-time among all the collaborators. This means users not only generate reconstructions on their own, but can also edit the reconstructions created by others as well, e.g. deleting a neurite, changing the local geometry of a neurite, etc. Moreover, the collaboration is flexible in that users can even work at different regions of the whole-brain imaging data at different level-of-details.

Supplementary References

1. Peng, H., Ruan, Z., Long, F., Simpson, J. H. & Myers, E. W. V3D enables real-time 3D visualization and quantitative analysis of large-scale biological image data sets. *Nature Biotechnology* **28**, 348–353 (2010).
2. Stefani, C., Lacy-Hulbert, A. & Skillman, T. ConfocalVR: Immersive Visualization for Confocal Microscopy. *Journal of Molecular Biology* **430**, 4028–4035 (2018).
3. Usher, W. *et al.* A Virtual Reality Visualization Tool for Neuron Tracing. *IEEE Transactions on Visualization and Computer Graphics* **24**, 994–1003 (2018).
4. Peng, H., Bria, A., Zhou, Z., Iannello, G. & Long, F. Extensible visualization and analysis for multidimensional images using Vaa3D. *Nature Protocols* **9**, 193–208 (2014).
