# Supplementary material for: TeraVR empowers precise reconstruction of complete 3-D neuronal morphology in the whole brain
Source: Nat Commun. 2019 Aug 2;10:3474. doi: 10.1038/s41467-019-11443-y (PMC6677772; doi:10.1038/s41467-019-11443-y)
Supplement: Supplementary file 2 — Description of Additional Supplementary Files [file 41467_2019_11443_MOESM2_ESM.pdf]

Description of Additional Supplementary Files

File name: Supplementary Movie 1
Description: Zoom in and zoom out functions of TeraVR.

File name: Supplementary Movie 2
Description: Translation / shift to an adjacent image area in TeraVR.

File name: Supplementary Movie 3
Description: An example of neurite-tracing using TeraVR.

File name: Supplementary Movie 4
Description: Examples of neurite-deletion and undo/redo operations in TeraVR.

File name: Supplementary Movie 5
Description: Addition and removal of markers in TeraVR.

File name: Supplementary Movie 6
Description: Examples of splitting and subdividing of a tract and dragging nodes (control points) of a tract in TeraVR.

File name: Supplementary Movie 7
Description: An example of adjusting contrast in TeraVR.

File name: Supplementary Movie 8
Description: Examples of displaying and hiding of annotations in TeraVR.

File name: Supplementary Movie 9
Description: An example of changing colors of annotations in TeraVR.

File name: Supplementary Movie 10
Description: An example of switching image channels in TeraVR.

File name: Supplementary Movie 11
Description: Automatic rotation of an image volume in TeraVR.

File name: Supplementary Movie 12
Description: A mixed-reality based demonstration on how TeraVR works.
